# Engineering Achiral Liquid Crystalline Polymers for Chiral Self-Recovery

**DOI:** 10.3390/ijms222111980

**Published:** 2021-11-05

**Authors:** Tengfei Miao, Xiaoxiao Cheng, Yilin Qian, Yaling Zhuang, Wei Zhang

**Affiliations:** State and Local Joint Engineering Laboratory for Novel Functional Polymeric Materials, Jiangsu Engineering Laboratory of Novel Functional Polymeric Materials, College of Chemistry, Chemical Engineering and Materials Science, Soochow University, Suzhou 215123, China; mtf0824@163.com (T.M.); studentscheng@163.com (X.C.); ylqian@stu.pku.edu.cn (Y.Q.); sa21175113@mail.ustc.edu.cn (Y.Z.)

**Keywords:** chiral induction, chiral self-recovery, supramolecular chirality, chiral nematic phase

## Abstract

Flexible construction of permanently stored supramolecular chirality with stimulus-responsiveness remains a big challenge. Herein, we describe an efficient method to realize the transfer and storage of chirality in intrinsically achiral films of a side-chain polymeric liquid crystal system by combining chiral doping and cross-linking strategy. Even the helical structure was destroyed by UV light irradiation, the memorized chiral information in the covalent network enabled complete self-recovery of the original chiral superstructure. These results allowed the building of a novel chiroptical switch without any additional chiral source in multiple types of liquid crystal polymers, which may be one of the competitive candidates for use in stimulus-responsive chiro-optical devices.

## 1. Introduction

Chirality, a fundamental property of nature, usually plays an essential role in biological processes and living systems. Nature is expert in using simple building blocks to efficiently fabricate highly complex systems, as well as the elegant assembly that can exhibit a variety of excellent performance [1,2]. Inspired by this, research on the transfer, amplification and memory of chirality between multi-levels, including secondary, tertiary and quaternary structures, is of key importance for understanding the origin of homochirality, as well as for the possible development of novel chiral materials [3,4,5].

Manipulating and memorizing hierarchically assembled chiral structures in achiral matters is an everlasting curiosity for scientists due to the avoidance of expensive chiral raw materials or tedious synthesis [6,7,8,9,10]. Meanwhile, chirality transfer from the chiral additive to achiral polymer chains via non-covalent interactions is a vital respect for propelling their applications in chiral sensors, chiroptical switches, chiral recognition and asymmetric catalysis [11]. Up-to-date, chiral solvation [12,13,14], circularly polarized light (CPL) [15,16,17], chiral liquid crystal field [18,19] and chiral template/scaffold [20,21,22] are candidates to generate macromolecular or supramolecular chirality in achiral polymer systems. However, the chiral supramolecular structures formed by non-covalent interactions between achiral building blocks are often easily destroyed by external stimulus [23,24,25], which undoubtedly limits the application of chiral materials. Typically, the chirality cannot be restored in the absence of pre-chiral resources. It can be speculated that constructing permanently memorized chirality with superior controllability or self-recovery capability in an achiral polymer system remains an extreme challenge. To achieve this, the adoption of covalent fixation after self-assembly via cross-linking of polymer chains will be a good choice [26].

The fascinating helical superstructure in the liquid crystal (LC) state is a striking example that reveals the structure–property relationship [27,28]. Similarly, introducing mirror symmetry breaking into LCs will commonly result in multitudinous chiral LC phases, such as the chiral nematic (N*) [29,30], chiral smectic (S*) [31], twist grain boundary (TGB) [32] and cubic blue phases [33], which have been well investigated in small molecular LCs. As reported by Li et al. [34,35,36], the sign and the magnitude of the N*-LCs pitches are strongly governed by chiral dopants due to their good fluidity. From another point of view, good fluidity is often accompanied by poor stability and a strong dependence on chiral resources. Among the self-organized supramolecular systems, chiral liquid crystalline polymers (LCPs) represent a promising class of materials that might exhibit stable supramolecular helical organizations [37,38]. Usually, the induced chirality can only be temporarily stored in the achiral systems through the interactions between polymer chains but will be completely destroyed when undergoing a phase transition [39]. Combining the cross-linking strategy and self-organization of achiral mesogens in side-chain liquid crystalline polymers, we set out to develop a novel, controllable helical structure where the induced chiral information can be stored permanently and be able to spontaneously trigger the self-recovery of original chiral superstructures even after being completely destroyed.

We have previously reported the induction and storage of supramolecular chirality via chiral limonene vapor fumigation and cross-linking in achiral polymer chains containing π-π conjugated azobenzene groups [40]. However, whether the supramolecular chirality can be induced and stored in the muti-types of liquid crystalline polymers such as the polymer containing non-coplanar mesogens cyanobiphenyl (CNB) or the phenyl benzoate (PB) group remains to be explored. Moreover, dynamically controlling the chiral signal intensity and the “on-off” chiroptical switch in the above polymer systems has rarely been achieved.

## 2. Results and Discussion

**Figure 1:**
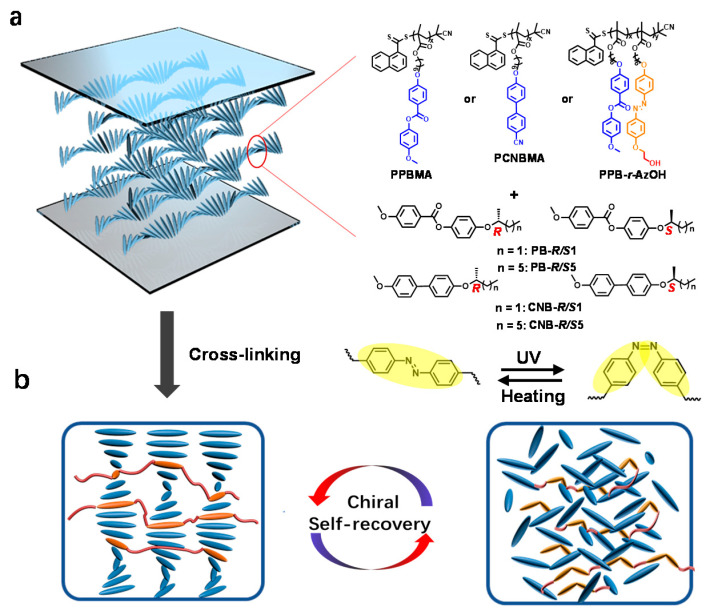
(**a**) Schematic representation of the chiral nematic phase formed from the achiral polymers and chiral dopants. (**b**) Process of the locking and self-recovery of the induced chirality.

In the current work, a series of liquid crystalline polymers were synthesized with controlled molecular weights (M_n_s) and molecular weight distributions via reversible addition-fragmentation chain transfer (RAFT) polymerization. The non-coplanar mesogens, cyanobiphenyl (CNB) or phenyl benzoate (PB) groups were introduced into the polymer as side chains for chiral self-organization. Besides, the hydroxyl-azobenzene, the photosensitive cross-linkable LC group [41,42], was also adopted to allow the regulation and permanent memory of the induced helical superstructure. The structures of the achiral polymers (PCNBMA_1–5_, PPBMA_1–5_ and PPBMA-r-AzOH) and chiral dopants are listed in Figure 1. The synthetic routes to the monomers (CNBMA, PBMA and AzOH) and polymers have been described in Scheme S1. As shown in Table 1 and Figures S1–S3, the polymers with different M_n_s were successfully prepared by changing the relative molar ratios of monomers and chain transfer agent (CTA). The small chiral molecules (PB-R/S1, PB-R/S5, CNB-R/S1 and CNB-R/S5) were successfully synthesized and determined by ^1^H NMR spectra and chiral high-performance liquid chromatography (HPLC) (Figures S4–S6).

First of all, the formation of the chiral nematic phase was investigated and determined using differential scanning calorimetry (DSC), small-angle X-ray scattering instrument (SAXS) and polarized optical microscope (POM) (Figures S7–S9). After being doped with a certain amount of chiral dopant, no scattering peak was observed both in the mixture of PPBMA/PCNBMA and PB-R1/CNB-R1 (Figure S8), indicating the absence of any layer structure corresponding to the smectic phase. Besides, the typical fingerprint textures of the chiral nematic phase were clearly observed when undergoing an appropriate annealing process (Figure S9). It can be speculated that the orientation of the mesogens in polymer side chains (PBs or CNBs) are slightly twisted with respect to their neighbors under the command of chiral dopants, leading to a long-range helical periodic superstructure [43].

**Figure 2:**
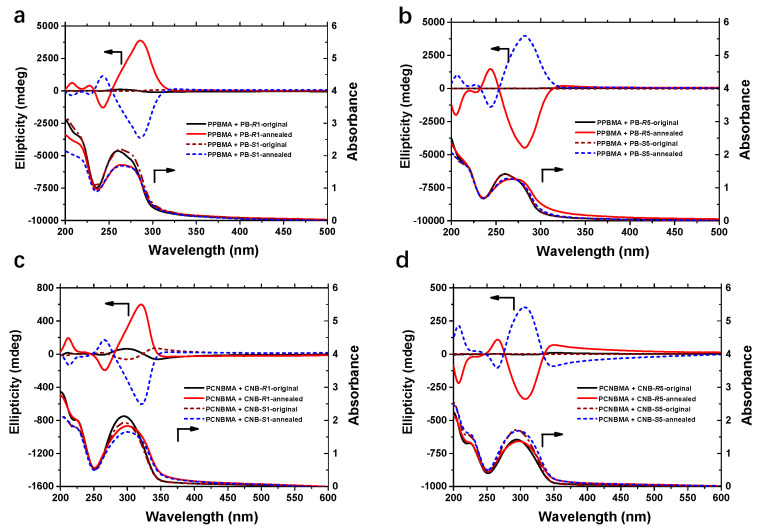
Chiral expression of the polymer films containing different components. (**a**) CD and UV-vis spectra of PPBMA_3_ doped with PB-*R/S*1. (**b**) CD and UV-vis spectra of PPBMA_3_ doped with PB-*R/S*5. (**c**) CD and UV-vis spectra of PCNBMA_3_ doped with CNB-*R/S*1. (**d**) CD and UV-vis spectra of PCNBMA_3_ doped with CNB-*R/S*5.

Chirality transfer from the chiral dopant to achiral polymers in solid-state film (prepared from THF solution containing achiral polymers and chiral dopants, as described in experimental methods) was then explored in detail by UV-vis and circular dichroism (CD) spectroscopy (Figure 2). The strong absorption bands from 230 nm to 300 nm and from 250 nm to 350 nm are attributed to the π-π* transition of the cyanobiphenyl and phenyl benzoate group, respectively. Originally, both the PPBMA and PCNBMA films exhibited a weak Cotton effect in the CD spectra when doped with chiral molecules. As expected, intense CD signals consisting of two overlapped exciton couplets that correspond to the π-π* transitions of PBs or CNBs units were clearly detected when increasing the temperature (Figure 2). A positive sign at the first Cotton band and a negative sign at the second Cotton band were observed when the PB/CNB-R1 was employed. Conversely, PB/CNB-S1 exhibited a mirror-image Cotton effect. It should be noted that the optically active polymer films show completely opposite Cotton effect when doped with PB/CNB-R5 or PB/CNB-S5 as compared with PB/CNB-R1 or PB/CNB-S1. We can speculate that the helical direction can be controlled not only by the configuration but also by the alkyl chain length of the chiral dopants.

**Figure 3:**
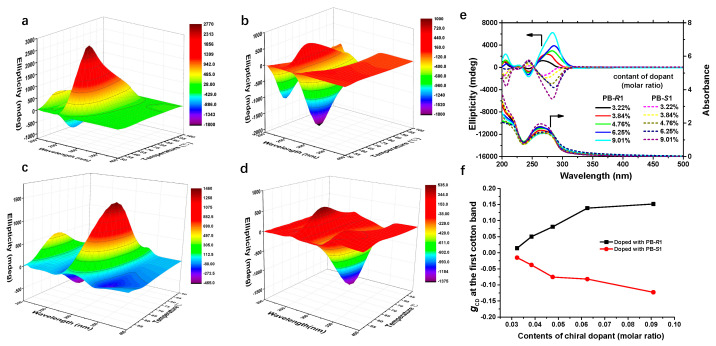
(**a**–**d**) CD spectra of the chiral induction of different polymer films during the heating process. (**a**) PPBMA_3_ film doped with PB-*R*1. (**b**) PPBMA_3_ film doped with PB-*S*1. (**c**) PCNBMA_3_ doped with CNB-*R*1. (**d**) PCNBMA_3_ doped with CNB-*S*1. (**e**) CD and UV-vis spectra of PPBMA_3_ doped with different content of PB-*R*1 and PB-*S*1. (**f**) *g*_CD_ values of PPBMA_3_ doped with different content of PB-*R*1 and PB-*S*1, calculated from (**e**).

Besides, the enhancement of CD signals indicates that the non-polar mesogens in the polymer side chains self-organized into a preferential chiral structure via cooperative interactions with the chiral dopants. The observed CDs are not the contribution of dopants in polymer film but mainly derived from the helical structure of polymers because no obvious CD decline can be found when chiral polymer films were immersed in methanol to remove the chiral dopants, as described in Section 3.3 and Figure S10. Temperature plays an important role during the chiral induction process due to the instability of the chiral nematic phase. The evolution of the CD spectra of polymer films during the heating process was tracked in detail. As shown in Figure 3a–d, CD intensity increased with increasing temperature until reaching their maximum values at around 72 °C (PPBMA films) and 80 °C (PCNBMA films). More prolonged heating of the polymer films resulted in a deep decrease in CD intensity ascribed to the fluidity of the nematic phase. Increasing the relative molar ratio of the dopant caused an enhancement in the intensity of ellipticity values (Figure 3e,f), which may be ascribed to the decrease in helical pitch (β = (pc)^−1^, β: helical twisting power of the dopant, p: helical pitch, c: the content of the dopant). The decrease in helical pitch means the increase of dihedral angles of two exciton couplets (much smaller than the extreme case, according to Nina’s exciton theory) [44].

**Figure 4:**
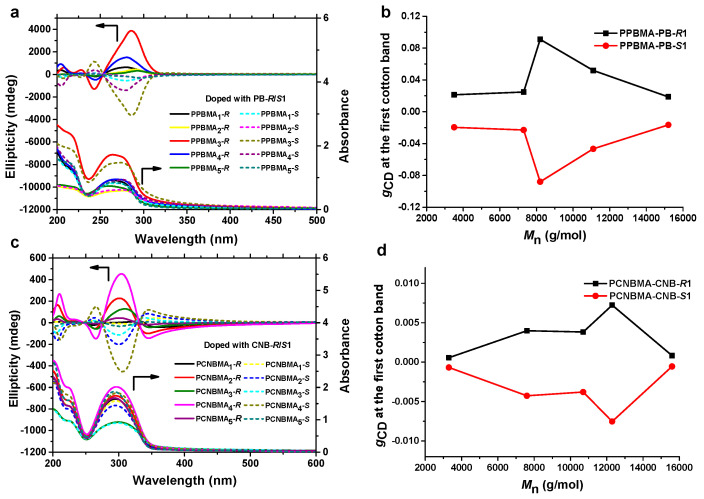
Changes in CD, UV-vis spectra and *g*_CD_ values of polymer films prepared from the polymers with different molecular weights. (**a**,**b**) recorded from PPBMAs. (**c**,**d**) recorded from PCNBMAs.

The polymer films containing PPBMA_1–5_ and PCNBMA_1–5_ with different M_n_s were prepared to investigate the effect of the polymer molecular weights on chiral induction. After chiral doping and heating–cooling treatment, the maximum g_CD_ values for each polymer film were recorded. As presented in Figure 4, the absolute maximum g_CD_ values of PPBMAs or PCNBMAs first increased and then decreased with the molecular weight after reaching their maximum values (PPBMA_3_, 8200 g/moL and PPBMA_4_, 12,300 g/moL), respectively.

**Figure 5:**
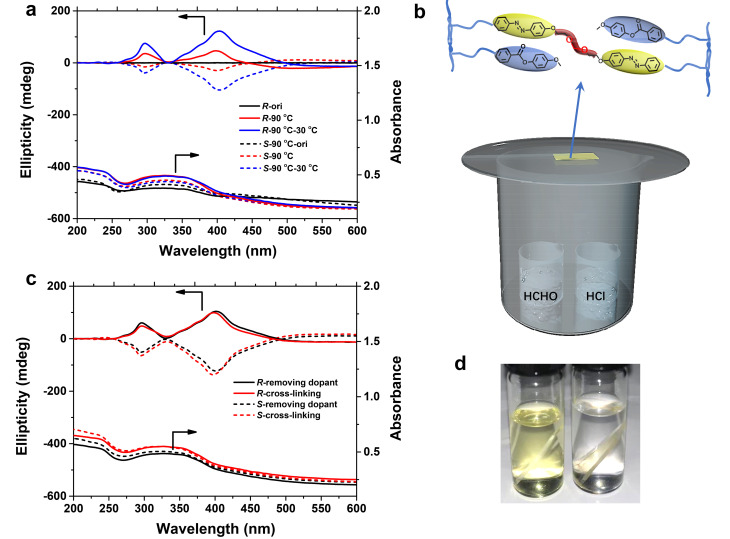
(**a**) CD and UV-vis spectra of the copolymer (PPB-*r*-AzOH) films doped with PB-*R*/*S*1. (**b**) Schematic diagram of the reaction method during the cross-linking process. (**c**) CD and UV-vis spectra of the chiral films after removing chiral dopants and after cross-linking reaction. (**d**) Pictures of the chiral polymer films placed in THF solution (left, before cross-linking; right: after cross-linking).

In order to flexibly control and permanently store the induced supramolecular chirality in the achiral polymer systems, photoisomerizable azobenzene units containing hydroxyl at a terminal for covalent cross-linking were introduced into the polymer side chain. Firstly, the chiral order of PPB-r-AzOH induced by chiral dopant was studied by CD spectra. After heating the polymer film (doped with PB-R1) to 90 °C, a positive sign of the first Cotton band at 400 nm appeared, which is due to the π-π* transitions of chirally arranged E-azobenzene units. However, no exciton couplet center was seen in the CD spectrum, and the second positive and weaker Cotton band at around 280 nm was found (Figure 5a). This is caused by the overlapping between the second weaker Cotton band belonging to azobenzene (negative) and the first stronger Cotton band belonging to phenyl benzoate (positive). Mirror-image Cotton effects were observed in the case of doping with PB-S1. Then the polymer film was immersed in methanol to remove the chiral dopant (see Section 3.3). The removal of chiral dopant from polymer film was confirmed by ^1^H NMR and HPLC spectra. As shown in Figure S11, the complete disappearance of the dopant signal indicates that the small chiral molecules have been removed successfully. The immersing process had no obvious effect on the CD spectra (Figure S14a). After chiral induction and the removal of the chiral source, the copolymer films were placed into an HCHO and HCl vapor atmosphere for covalent cross-linking via acetal reaction (Figure 5b). Differences in solubility of polymer films before and after cross-linking in THF revealed the success of cross-linking reactions (Figure 5d). Importantly, the chiral structure was perfectly preserved throughout the cross-linking procedure, as confirmed by the almost identical ellipticity values in CD spectra (Figure 5c).

**Figure 6:**
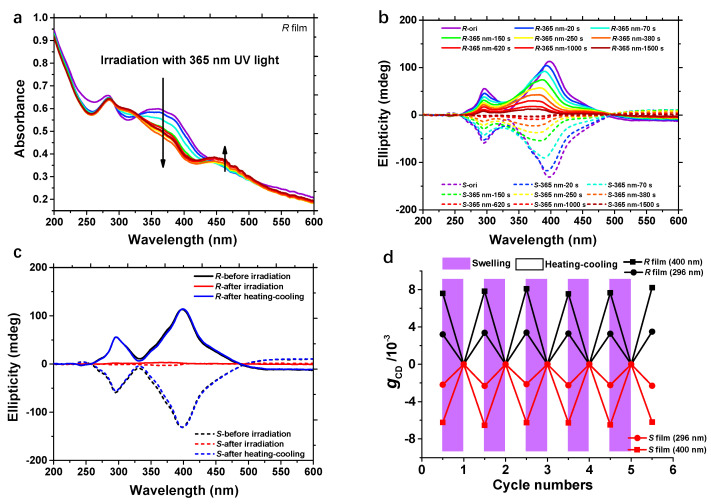
(**a**) Changes in UV-vis spectra of the cross-linked chiral polymer films under irradiation with 365 nm light. (**b**) Changes in CD spectra of the cross-linked chiral polymer films (induced by PB-*R*/*S*1) during 365 nm light irradiation. (**c**) Self-recovery of CD signals of the irradiated chiral films during the heating–cooling process. (**d**) Construction of chiroptical switch without chiral resource: destruction and chiral self-recovery behavior: switches of the cross-linked films during the above process (as shown by *g*_CD_ values, calculated from Figure S15).

Distinct from uncross-linked counterparts, the cross-linked PPBMA films exhibited superior thermal stability even in the absence of chiral dopant. When heating the film to a high temperature (105 °C), a slight decrease of CD intensity occurred ascribed to partial fluidity of mesogenic units. The shape of the CD spectra remained almost unchanged with extended heating time and recovered to the original state after cooling down the films to room temperature (Figure S12). We further investigated the chiral self-recovery behavior of the supramolecular structure upon exposure to UV light irradiation. As reported previously, the trans forms of some Azo derivatives exhibited a nematic phase, whereas their cis forms showed no LC property [45]. For chiral PPB-r-AzOH films, UV-vis spectra showed a clear decline at 365 nm (π-π* electronic transition of the trans isomers) and an increase at 450 nm (n-π* electronic transition of the cis isomers) upon irradiation with 365 nm light, demonstrating the trans−cis photoisomerization of the Azo groups (Figure 6a). After 1500 s of irradiation, the regular helical structures of both the phenyl benzoate and azobenzene portion were completely disrupted, as confirmed by the disappearance of the CD signals (Figure 6b). Traditionally, the presence of a chiral source is necessary both in solution or solid films for the “on-off” chiroptical switch [46,47]. Interestingly, as displayed in Figure 6c, the shapes and intensities of CD spectra can return to their original states by a simple heating–cooling treatment (heated to 95 °C, maintained for 3 min before cooling down to room temperature). The uncross-linked chiral polymer films will lose the chiral information when they are irradiated by UV light after removing the dopants s (Figure S14). It can be speculated that the cross-linked organized Azo units can provide cryptochirality to trigger the complete self-recovery of the entire chiral superstructure. This means that the chiral information from an external source can be permanently memorized in achiral polymer systems.

The reversible chiral–achiral switching can enhance the functionality of optically active materials. Indeed, the traditional approach always involves sophisticated design and noticeable dependence on the chiral source. Combining the chiral doping and cross-linking strategy, a chiroptical switch based on the absolute achiral polymer can be successfully constructed. The above “on-off” switching could be repeated at least five times without any noticeable loss in CD intensity (Figure 6d and Figure S15).

## 3. Materials and Methods

### 3.1. Materials

Chiral alcohols including (R)-(-)-butanol (>99.0%, TCI), (S)-(+)-butanol (>98.0%, TCI), (R)-(-)-octanol (>99%, TCI) and (S)-(+)-octanol (>99%, TCI) were used without further purification. Moreover, 6-Chlorohexyl methacrylate, 4-(2-hydroxy ethoxy)-4′-(2-hexyloxy methacrylate) azobenzene (AzOH) were synthesized as reported previously [40]. The compounds 4-methoxyphenyl 4-hydroxybenzoate, 4-cyano-4 -hydroxybiphenyl and 4-hydroxy-4-methoxybiphenyl were purchased from Sigma-Aldrich (St. Louis, MO, USA). The preparation steps for achiral monomers PBMA, CNBMA, chiral dopants and liquid crystalline polymers were listed in supplementary materials.

### 3.2. Preparation of the Chiral Polymer Films

The achiral polymer films for CD measurements with thickness ranging from 100 to 200 nm were prepared by spin coating the polymer solution (12 mg/mL in THF, doped with a certain number of chiral molecules) onto a clean quartz plate at a speed of 0.5 rpm for 6 s and following a speed of 1.9 rpm for 20 s. After drying under vacuum to remove THF, the film was heated to the nematic temperature to ensure the occurrence of chiral transmission.

### 3.3. Removal of Chiral Dopants

The chiral polymer film containing chiral dopants was immersed in methanol for 50 h to remove the chiral dopants, during which the immersing liquid was changed at least six times. After that, the residual amount of chiral dopant in polymer films was measured by ^1^H NMR and HPLC spectroscopy (Figure S11).

### 3.4. The Storage of the Supramolecular Chirality

After chiral induction and removal of the dopants, the chiral copolymer PPB-r-AzOH films were placed on the top of a larger beaker in which two small beakers containing 30% formaldehyde aqueous and 6 N hydrochloric acid solution were placed separately (Figure 5b). Then the cross-linking of the polymer chains was carried out during the exposure to HCl and HCHO vapor at 25 °C for 20 h. Finally, the cross-linked polymer films were washed with pure water to remove the residual HCl and HCHO molecules and then dried under vacuum at 35 °C for 12 h.

### 3.5. Photoisomerization Process of Polymer Films

Photoisomerization of cross-linked chiral copolymer film was conducted with a 500 W high-pressure mercury lamp (Tokyo, Japan), Optiplex SX-UID and 502HUV) equipped with a narrow bandpass filter for the wavelength of 365 nm. The intensity of UV light irradiation is 2.5 mW cm^−2^.

### 3.6. Characterization

^1^H NMR spectra were recorded on a Bruker nuclear magnetic resonance instrument (300 MHz, Brucker, Karlsruhe, Germany) using CDCl_3_ and DMSO-d_6_ as solvent and tetramethylsilane (TMS) as the internal standard at room temperature.

Gel-permeation chromatography (GPC) measurements were conducted on the TOSOH HLC-8320 gel permeation chromatography (GPC) (TOSOH, Japan), equipped with refractive index and UV detectors using two TSKgel SuperMultiporeHZ-N (4.6 × 150 mm, 3.0 μm beads size) columns arranged in series. Polymers can be separated in the molecular weight range of 500–190k Da. THF was used as the eluent with a flow rate of 0.35 mL/min at 40 °C. The number average molecular weights (M_n_) and molecular weight distributions (*M*_W_/*M*_n_) of samples were calculated according to polystyrene (PS) standards.

The phase transition of the polymers was measured with a TA-Q100 DSC instrument (TA, New Castle, DE, USA). The temperature range is 20 °C to 200 °C with the first heating–cooling speed of 20 °C/min and second heating speed of 10 °C/min.

LC textures and birefringence of the samples were examined under a POM (Olympus Corporation, BX51-P, Japan) equipped with a hot stage (Linkam THMS600).

SAXS experiments were performed with a high-flux X-ray instrument (SAXSess mc^2^, Anton Paar, Austria) equipped with a line collimation system and a 2200 W sealed-tube X-ray generator (CuKα, λ = 0.154 nm). The polymer samples were wrapped in aluminum foils and sandwiched in a steel sample holder.

The CD spectra were recorded on a JASCO J-1500 spectropolarimeter equipped with a Peltier-controlled housing unit using an SQ-grade cuvette, a single accumulation, a bandwidth of 2 nm, a scanning rate of 200 nm/min, a path length of 10 mm and a response time of 1 s. The magnitude of the circular polarization at the ground state is defined as g_CD_ = 2 × (ε_L_ − ε_R_)/(ε_L_ + ε_R_), where ε_L_ andε_R_ denote the extinction coefficients for left and right circularly polarized light, respectively. Experimentally, g_CD_ value is defined as Δε/ε = [ellipticity/32,980]/absorbance at the CD extremum. The UV-vis spectra were recorded on a UV-2600 spectrophotometer (Shimadzu (Nakagyo-ku, Kyoto, Japan)).

Chiral HPLC was performed on an Agilent 1200 Series chromatography (Agilent, California USA) using a Daicel Chiralpak AD-H column (0.46 cm × 25 cm).

## 4. Conclusions

In summary, we have demonstrated a novel approach to construct an optically active polymer film with adjustable and permanently memorized supramolecular chirality via chiral doping and cross-linking. Side-chain liquid crystalline polymers containing non-coplanar mesogens CNB or PB were adopted as achiral hosts. We have investigated the influence of temperature, molecular weight, dopant structure and contents on the chiral expression in detail. Furthermore, introducing cross-linkable azobenzene units allows the photo regulation and storage of the induced chirality, which enables the construction of a chiroptical switch in the absence of chiral sources. Our research will pave a novel and efficient way for the design and construction of functional chiral polymer materials with reversible chiroptical properties from absolute achiral building blocks.

## Figures and Tables

**Table 1:** Molecular weight characteristics of liquid crystalline polymers.

Entry	Ratio ^1^	Conv. ^2^ (%)	*M*_n(GPC)_ ^3^ (g/mol)	*M*_w_/*M*_n_^3^
PPBMA_1_	40:3:1	59.7	3500	1.17
PPBMA_2_	100:3:1	50.2	7300	1.12
PPBMA_3_	200:3:1	41.6	8200	1.23
PPBMA_4_	300:3:1	32.9	11,100	1.21
PPBMA_5_	500:3:1	22.7	15,200	1.17
PPB-*r*-AzOH	60:40:3:1	46.6	8800	1.14
PCNBMA_1_	40:3:1	61.6	3300	1.12
PCNBMA_2_	100:3:1	56.3	7600	1.18
PCNBMA_3_	200:3:1	40.9	10,700	1.14
PCNBMA_4_	300:3:1	29.3	12,300	1.20
PCNBMA_5_	500:3:1	21.3	15,600	1.21

^1^ Polymerization conditions for PPBMA_1–5_ and PCNBMA_1–5_: [monomer]_0_/[CPDN]_0_/[AIBN]_0_. Polymerization conditions for PPB-*r*-AzOH: [PBMA]_0_/[AzOH]_0_/[CPDN]_0_/[AIBN]_0_). ^2^ Determined gravimetrically. ^3^ Determined by GPC according to PS standards in THF.

## Data Availability

No datasets have been used for this study.

## Supplementary Materials

The following are available online at https://www.mdpi.com/article/10.3390/ijms222111980/s1.
