# Supplementary material for: Engineering Achiral Liquid Crystalline Polymers for Chiral Self-Recovery
Source: Int J Mol Sci. 2021 Nov 5;22(21):11980. doi: 10.3390/ijms222111980 (PMC8584346; doi:10.3390/ijms222111980)
Supplement: Supplementary file 1 [file ijms-22-11980-s001.zip › ijms-1433401-supplementary.pdf]

Supplementary Materials

Engineering Achiral Liquid Crystalline Polymers for Chiral Self-Recovery

Tengfei Miao, Xiaoxiao Cheng, Yilin Qian, Yaling Zhuang and Wei Zhang*

Table of Contents

1. Materials and Synthesis……………………………………………………………S1

1.1 Synthesis of the achiral monomers……………………………………………S1

1.2 Synthesis of the achiral polymers……………………………………………S3

1.3 Synthesis of the chiral dopants………………………………………………S3

1.4 Structure information of the monomers, polymers and chiral dopants……………S4

2. Supporting Figures……………………………………………………………S7

3. References……………………………………………………………………S10

1. Materials and Synthesis

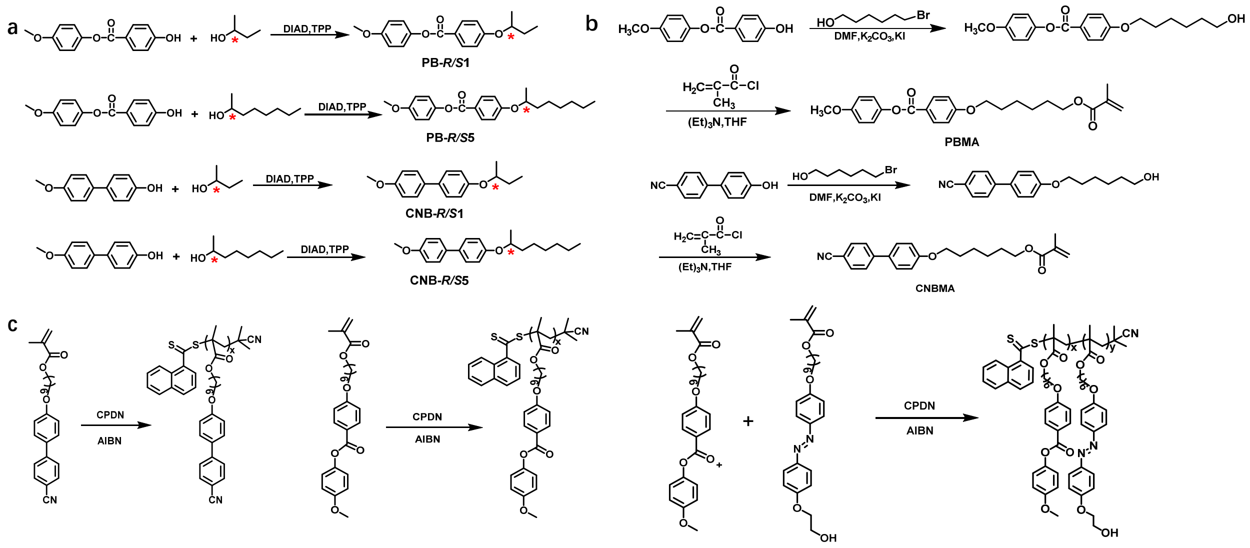

Scheme S1 Synthetic routes to the chiral dopants (a), monomers PBMA and CNBMA (b) and polymers (c).

1.1 Synthesis of the achiral monomers (Scheme S1a)

6-Chlorohexyl methacrylate and (2-hydroxy ethoxy)-4'-(2-hexyloxy methacrylate) azobenzene (AzOH) were synthesized according to the literature [1].

4-Methoxyphenyl 4-((6-(methacryloyloxy)hexyl)oxy)benzoate (PBMA) was synthesized via the following procedures. The compound 4-methoxyphenyl 4-hydroxybenzoate (2.83 g, 11.6 mmol), potassium carbonate (6.4 g, 46.5 mmol), a small amount of potassium iodide and 30 mL DMF were mixed into a 100 mL round bottom flask. The reaction mixture was refluxed under argon at 80 °C for 1 h. After that a solution of 6-Chlorohexyl methacrylate (3.21 g, 15.7 mmol) in 15 mL DMF was then added dropwise to the above solution. Then the reaction was carried out at 80 °C for 12 h. Finally, the reaction mixture was cooled down to room temperature and then poured into 200 mL water. The product was collected by filtration and purified by column chromatography with hexane/ethyl acetate (5:1, v/v). Finally, the white crystal product was obtained by recrystallization purification in hexane (2.92 g, 61.1% yield). ^{1}H NMR (DMSO-d_6, 300 MHz) of PBMA, (δ, ppm): 8.05 (m, 2H), 7.16 (m, 4H), 6.99 (m,

2H), 6.03 (d, 1H), 5.68 (d, 1H), 4.12 (m, 4H), 3.77 (s, 3H), 1.87 (s, 3H), 1.72 (m, 4H), 1.43 (m, 4H).

6-((4'-Cyano-[1,1'-biphenyl]-4-yl)oxy)hexyl methacrylate (CNBMA) was synthesized according to the similar process. ^{1}H NMR (DMSO-d_6, 300 MHz) of CNBMA, (δ, ppm): 7.85 (m, 4H), 7.70 (m, 2H), 7.05 (m, 2H), 6.01 (d, 1H), 5.62 (d, 1H), 4.13 (m, 2H), 4.03 (m, 2H), 1.90 (s, 3H), 1.81 (m, 2H), 1.71 (m, 2H), 1.48 (m, 4H).

1.2 Synthesis of the achiral polymers (Scheme S1c)

The side-chain liquid crystalline polymers with different M_ns were synthesized by reversible addition fragmentation chain transfer (RAFT) polymerization. CPDN and AIBN were used as the RAFT agent and the initiator, respectively. For the homopolymer $PPBMA_2$, the monomer PBMA (412 mg, 1.0 mmol), CPDN (8.35 mg, 0.03 mmol), AIBN (1.64 mg, 0.01 mmol) and dried anisole (1.5 mL) were added into a 5 mL ampoule bottle. The polymerization ratio is 100/3/1 ($[monomer]_0/[CPDN]_0/[AIBN]_0$). The reaction tube was flame-sealed under an argon atmosphere after deoxygenated with three freeze-thaw cycles. Then the polymerization was carried out at 70 °C for 3 h. After that, the reaction mixture was diluted with THF (2 mL) and precipitated into an excess of methanol (80 mL) twice. The polymer solid was collected by filtration and then dried in a vacuum oven overnight at 30 °C. Homopolymers PCNBMAs were synthesized via the similar process. Copolymer PPB-*r*-AzOH was also obtained via the similar process, except for the polymerization ratio of the monomers. The hydroxyl content was calculated from the ^{1}H NMR spectra (Figure S5).

1.3 Synthesis of the chiral compounds PB-*R*/*S*1, PB-*R*/*S*5, CNB-*R*/*S*1 and CNB-*R*/*S*5

Synthesis of **PB-*R*1**: 4-methoxyphenyl 4-hydroxybenzoate (488.0 mg, 2 mmol), (*R*)-(-)-2-octanol (260 mg, 2 mmol) and diisopropyl azodicarboxylate (0.61 mL, 3 mmol) were dissolved in 10 mL dried THF. The mixture was cooled in a water-ice bath for 0.5 h. Then the 5 mL THF solution containing triphenylphosphine (786.0 mg, 3 mmol) was added dropwise to the above mixture. After continuing stirring the liquid at 0 ºC for 30 min, the reaction was

carried out at room temperature for 12 h. After that, the precipitate was filtered off and the solvent was removed. The solid was washed with ether for at least three times. After concentrating the washing liquid, the product was further purified by column chromatography with hexane/ethyl acetate (15:1, v/v). Finally, the white crystal product was obtained by recrystallization purification in hexane. (443.5 mg, 62.3% yield). ^{1}H NMR (DMSO-d_6, 300 MHz) of the compound **PB-*R*1**, (δ, ppm): 8.04 (m, 2H), 7.15 (m, 2H), 7.09 (m, 2H), 6.98 (m, 2H), 4.59 (m, 1H), 3.78 (s, 3H), 1.62 (m, 2H), 1.27 (m, 3H), 0.94 (t, 3H). The other chiral dopants with different structure were prepared in similar procedures.

PB-*S*1, (δ, ppm): 8.05 (m, 2H), 7.17 (m, 2H), 7.07 (m, 2H), 7.00 (m, 2H), 4.59 (m, 1H), 3.78 (s, 3H), 1.67 (m, 2H), 1.27 (m, 3H), 0.92 (t, 3H).

PB-*R*5, (δ, ppm): 8.03 (m, 2H), 7.17 (m, 2H), 7.08 (m, 2H), 6.97 (m, 2H), 4.59 (m, 1H), 3.78 (s, 3H), 1.62 (m, 2H), 1.26 (m, 11H), 0.85 (t, 3H).

PB-*S*5, (δ, ppm): 8.05 (m, 2H), 7.15 (m, 2H), 7.07 (m, 2H), 6.98 (m, 2H), 4.59 (m, 1H), 3.78 (s, 3H), 1.59 (m, 2H), 1.26 (m, 11H), 0.85 (t, 3H).

CNB-*R*1, (δ, ppm): 7.53 (m, 4H), 6.95 (m, 4H), 4.40 (m, 1H), 3.77 (s, 3H), 1.62 (m, 2H), 1.24 (m, 3H), 0.92 (t, 3H).

CNB-*S*1, (δ, ppm): 7.50 (m, 4H), 6.96 (m, 4H), 4.39 (m, 1H), 3.78 (s, 3H), 1.64 (m, 2H), 1.24 (m, 3H), 0.94 (t, 3H).

CNB-*R*5, (δ, ppm): 7.50 (m, 4H), 6.96 (m, 4H), 4.44 (m, 1H), 3.76 (s, 3H), 1.58 (m, 2H), 1.25 (m, 11H), 0.85 (t, 3H).

CNB-*S*5, (δ, ppm): 7.51 (m, 4H), 6.96 (m, 4H), 4.44 (m, 1H), 3.76 (s, 3H), 1.60 (m, 2H), 1.25 (m, 11H), 0.85 (t, 3H).

1.4 Structure information of the monomers, polymers and chiral dopants

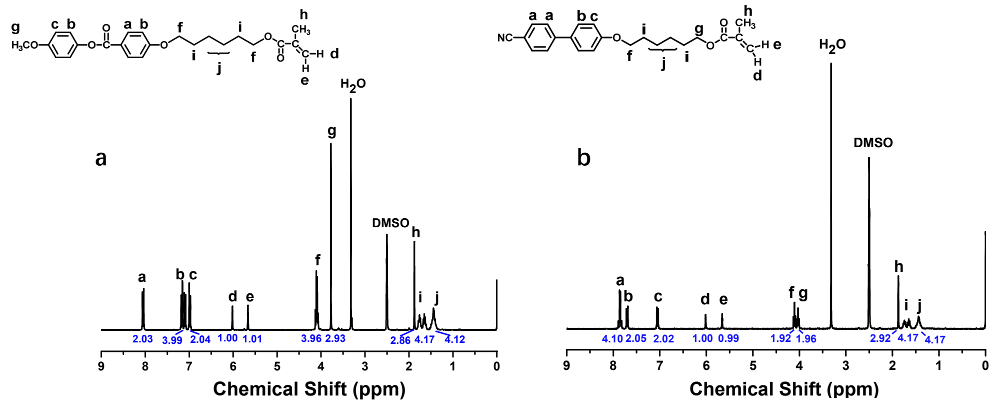

Figure S1 ^{1}H NMR spectra of the monomers PBMA (a) and CNBMA (b).

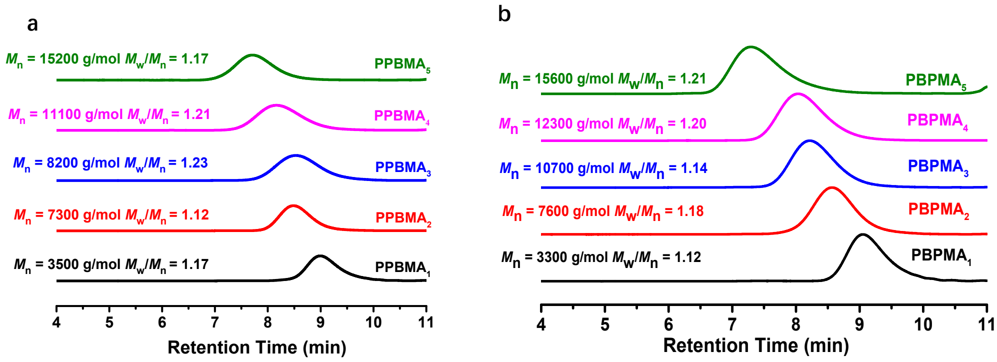

Figure S2 GPC curves of the homopolymers PPBMA1-5 (a) and PCNBMA1-5 (b).

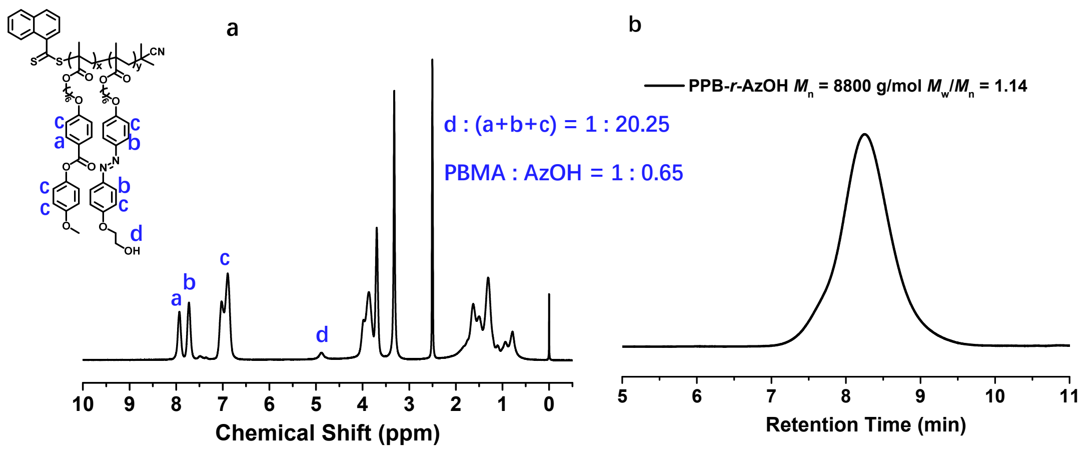

Figure S3 ^{1}H NMR spectra (a) and GPC curve (b) of the copolymer PPB-*r*-AzOH. The monomer ratio calculated by ^{1}H NMR is PBMA : AzOH = 1 : 0.65 (molar ratio).

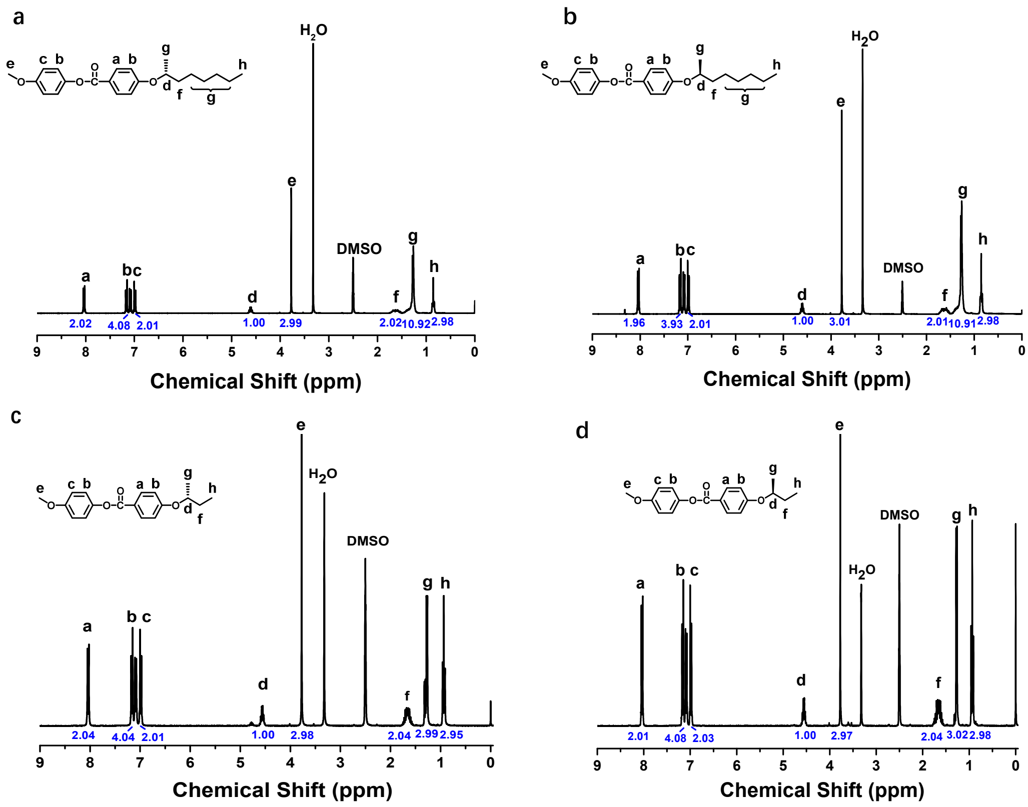

Figure S4 ^{1}H NMR spectra of the chiral dopants PB-*R*5(a), PB-*S*5(b), PB-*R*1(c) and PB-*S*1(d).

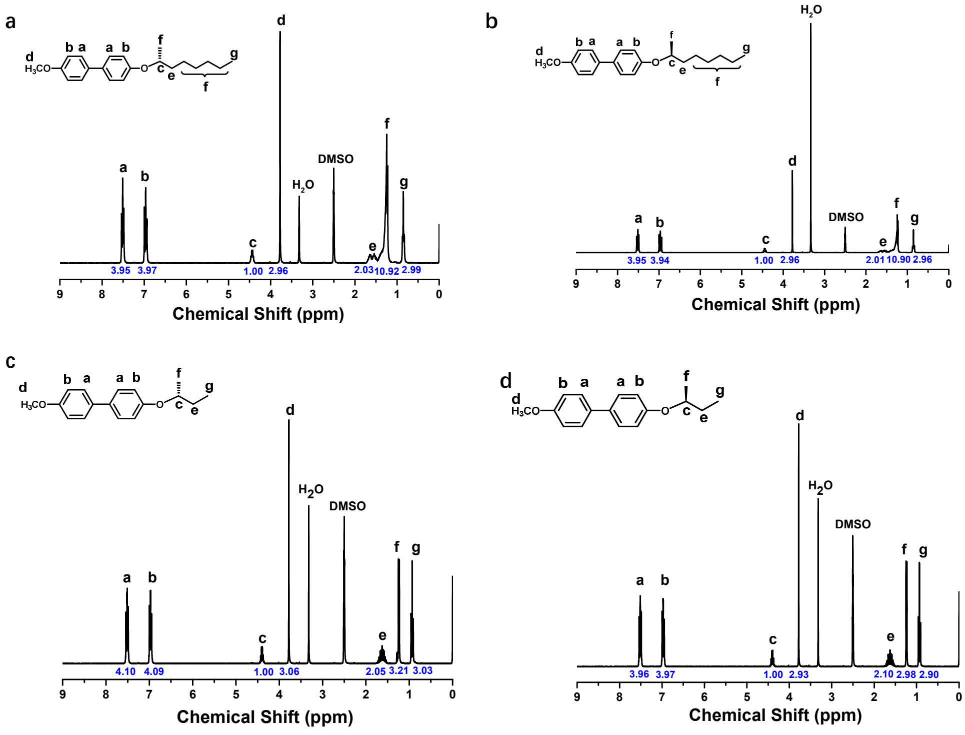

Figure S5 ^{1}H NMR spectra of the chiral dopants CNB-*R*5(a), CNB-*S*5(b), CNB-*R*1(c) and CNB-*S*1(d).

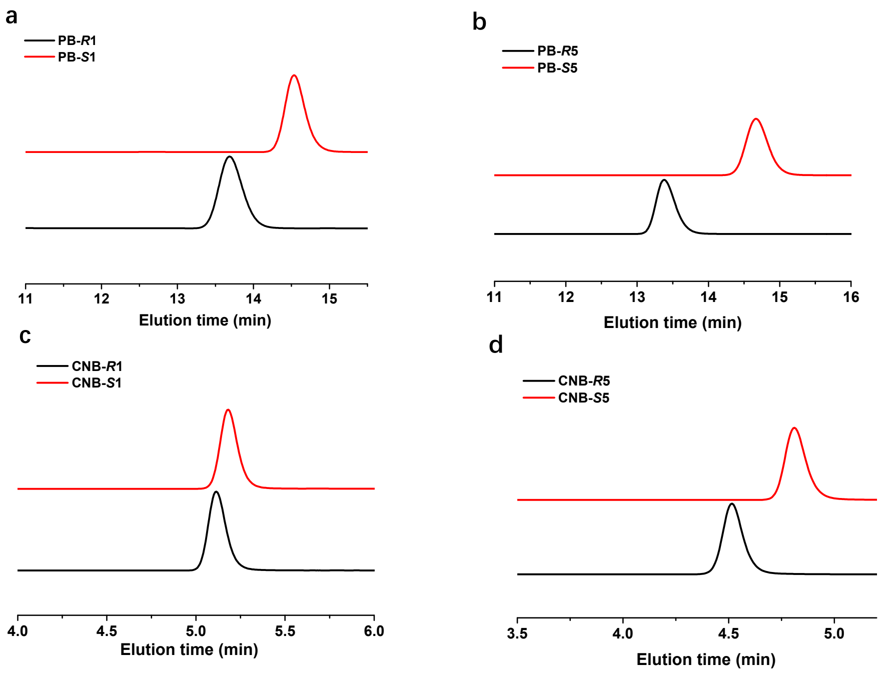

Figure S6 Chiral HPLC spectra of the chiral dopants.

2. Supporting Figures

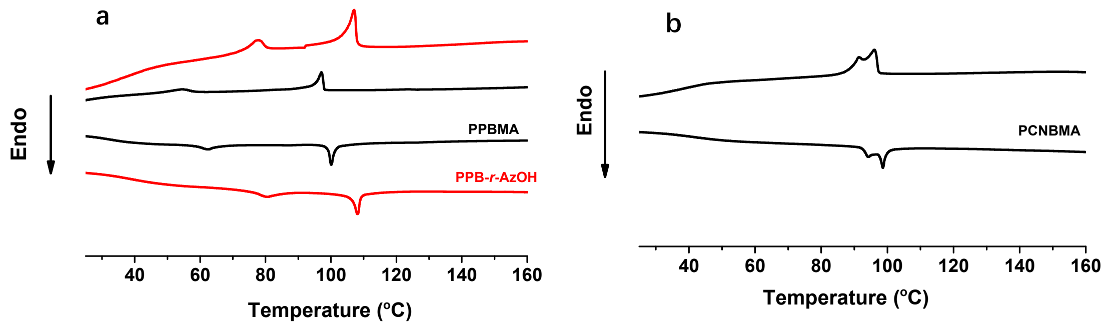

Figure S7 DSC curves of the polymers.

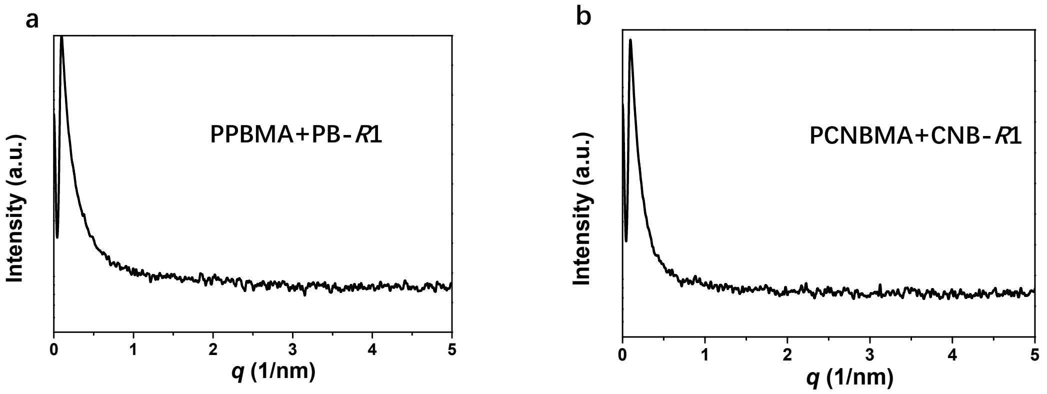

Figure S8 X-ray scattering profiles of PPBMA (a) and PCNBMA (b) solid doped with chiral molecules. The sample was heated above the clearing temperature and then annealed at 80 °C (nematic phase) for 12 h before measurements.

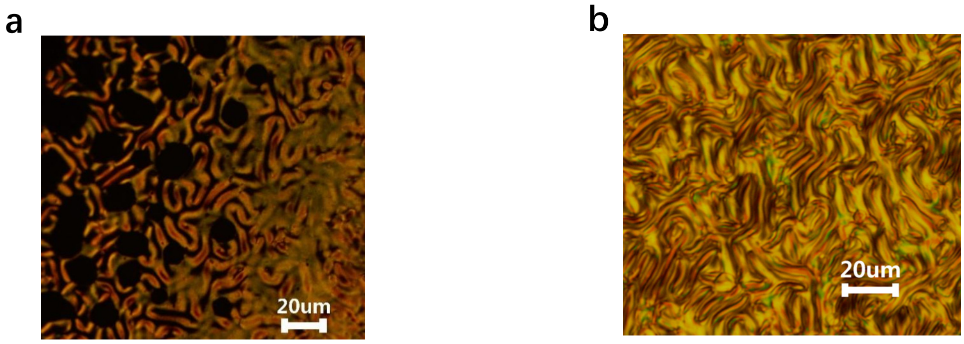

Figure S9 POM images of the chiral nematic phase of PPBMA (a) and PCNBMA (b) solid doped with chiral molecules. The mixture was annealed at 80 °C for 12 h after slowly cooled from isotropic state.

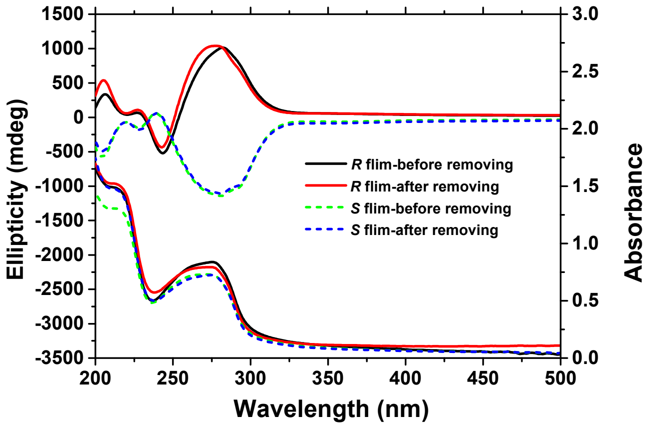

Figure S10 CD and UV-vis spectra of the chiral polymer films measured before and after removing the chiral dopants.

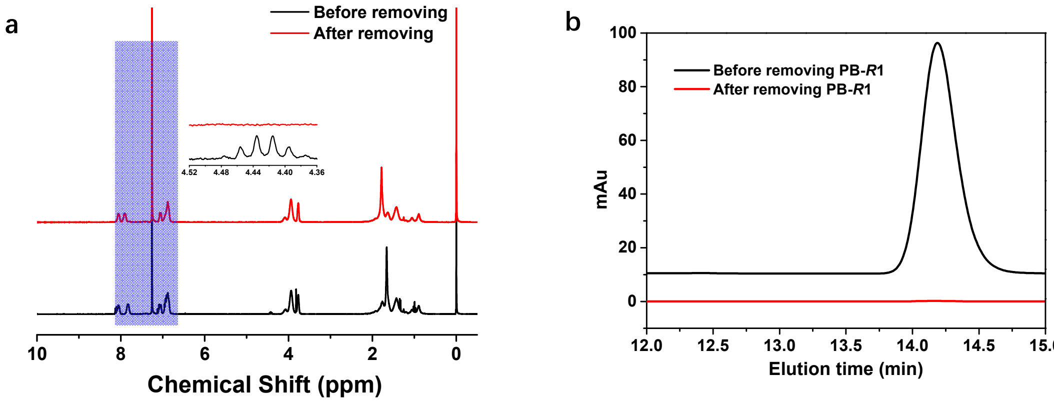

Figure S11 (a) ^{1}H NMR spectra of the chiral polymer film measured before and after removal of chiral dopant. (b) HPLC spectra of the chiral polymer film measured before and after removal of chiral dopant.

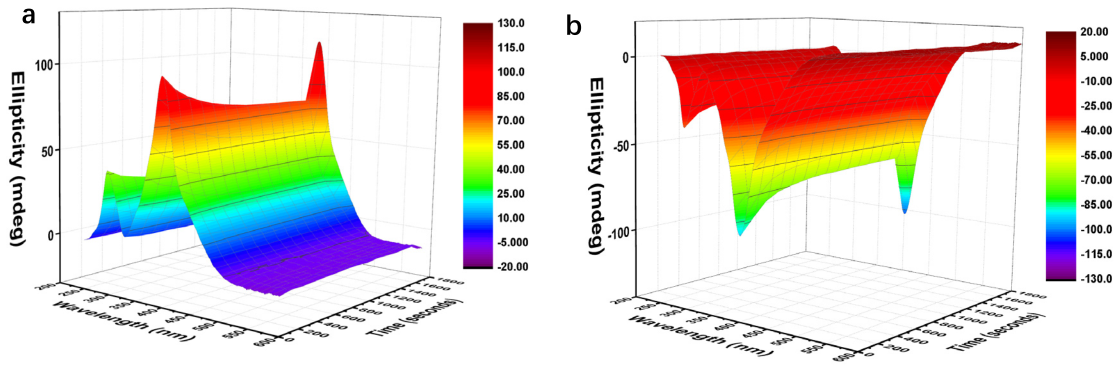

Figure S12 The CD spectra of the cross-linked chiral film measured at 105 °C.

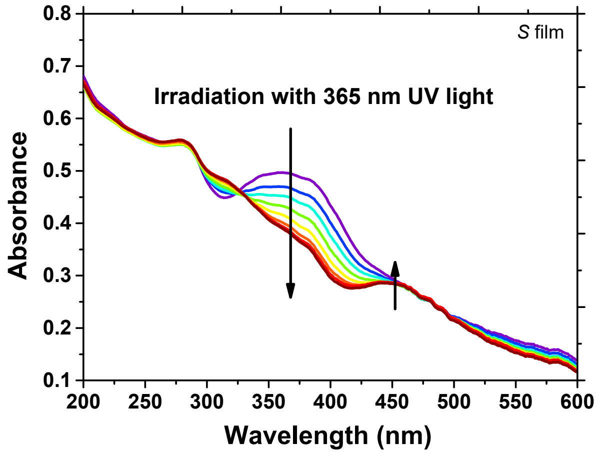

Figure S13 Changes in UV-vis spectra of the cross-linked chiral polymer films measured under irradiation with 365 nm light (*S* film).

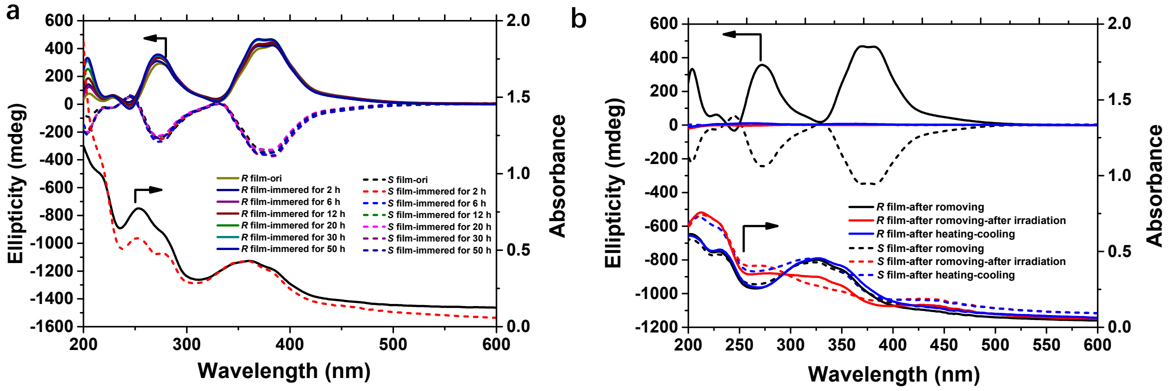

Figure S14. (a) Changes in the CD, UV-vis spectra of the uncross-linked chiral polymer films during the immersing process. (b) CD-clearing process of the uncross-linked chiral polymer films (after immersed) upon irradiation with 365 nm UV light.

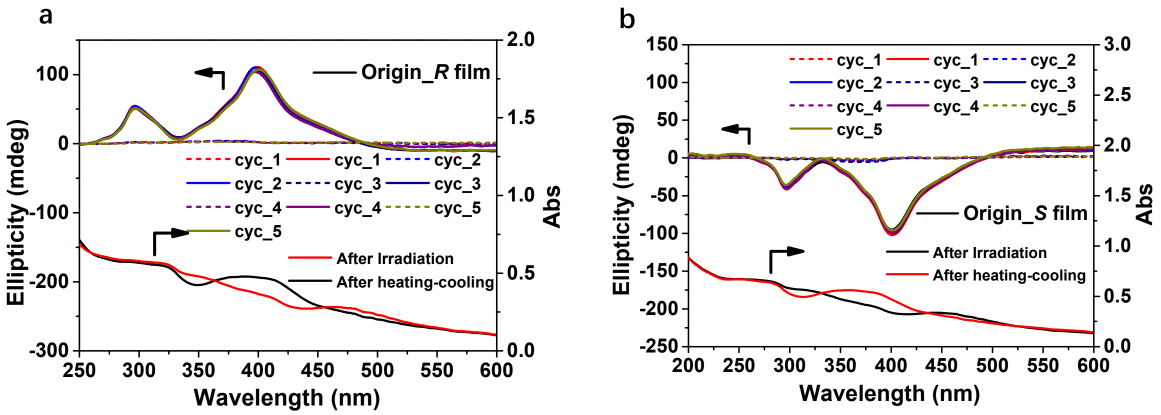

Figure S15 Changes in the CD and UV-vis spectra of the chiral polymer films (PPB-*r*-AzOH) in the switch of 365 nm light irradiation and heating-cooling process. The films are measured after removing the dopants and after cross-linking. (a) induced by PB-*R*1; (b) induced by PB-*S*1.

3. References

1. Miao, T.F.; Cheng, X.X.; Ma, H.T.; He, Z.X.; Zhang, Z.B.; Zhou, N.A.C.; Zhang, W.; Zhu, X.L. Transfer, amplification, storage, and complete self-recovery of supramolecular chirality in an achiral polymer system. *Angew. Chem. Int. Ed.* **2021**, *60*, 18566-18571.
